# Selection of Potential Yeast Probiotics and a Cell Factory for Xylitol or Acid Production from Honeybee Samples

**DOI:** 10.3390/metabo11050312

**Published:** 2021-05-13

**Authors:** Farah Zahoor, Chayaphathra Sooklim, Pattanan Songdech, Orawan Duangpakdee, Nitnipa Soontorngun

**Affiliations:** 1 Biochemical Technology Division, School of Bioresources and Technology, King Mongkut’s University of Technology Thonburi, Bangkok 10150, Thailand; farah.zahoor@yahoo.com (F.Z.); chayaphathra@gmail.com (C.S.); jup_freedom@hotmail.com (P.S.); 2 Native Honeybee and Pollinator Research Center, Ratchaburi Campus, King Mongkut’s University of Technology Thonburi, Rang Bua, Chom Bueng, Ratchaburi 70150, Thailand; orawan.dua@mail.kmutt.ac.th

**Keywords:** acid production, honey, functional food, probiotics, xylitol, yeast

## Abstract

Excessive use of antibiotics has detrimental consequences, including antibiotic resistance and gut microbiome destruction. Probiotic-rich diets help to restore good microbes, keeping the body healthy and preventing the onset of chronic diseases. Honey contains not only prebiotic oligosaccharides but, like yogurt and fermented foods, is an innovative natural source for probiotic discovery. Here, a collection of three honeybee samples was screened for yeast strains, aiming to characterize their potential in vitro probiotic properties and the ability to produce valuable metabolites. Ninety-four isolates out of one-hundred and four were able to grow at temperatures of 30 °C and 37 °C, while twelve isolates could grow at 42 °C. Fifty-eight and four isolates displayed the ability to grow under stimulated gastrointestinal condition, at pH 2.0–2.5, 0.3% (*w*/*v*) bile salt, and 37 °C. Twenty-four isolates showed high autoaggregation of 80–100% and could utilize various sugars, including galactose and xylose. The cell count of these isolates (7–9 log cfu/mL) was recorded and stable during 6 months of storage. Genomic characterization based on the internal transcribed spacer region (ITS) also identified four isolates of *Saccharomyces cerevisiae* displayed good ability to produce antimicrobial acids. These results provided the basis for selecting four natural yeast isolates as starter cultures for potential probiotic application in functional foods and animal feed. Additionally, these *S. cerevisiae* isolates also produced high levels of acids from fermented sugarcane molasses, an abundant agricultural waste product from the sugar industry. Furthermore, one of ten identified isolates of *Meyerozyma guilliermondiii* displayed an excellent ability to produce a pentose sugar xylitol at a yield of 0.490 g/g of consumed xylose. Potentially, yeast isolates of honeybee samples may offer various biotechnological advantages as probiotics or metabolite producers of multiproduct-based lignocellulosic biorefinery.

## 1. Introduction

Probiotics are microorganisms, usually called “helpful” or “good” bacteria or yeasts, that improve the gut biota and keep our gut healthy. As live microbial feed supplement, probiotics or their components beneficially affect the host health by improving the microbial balance of intestines [1]. On ingestion, they have an advantageous effect in the prevention and treatment of certain pathological conditions. As a matter of fact, probiotics have been used for as long as people have consumed foods like yogurt and other fermented nutriments [2] to cope with digestive problems such as disruption of the microbial balance, abnormal intestinal balance, nausea, vomiting, indigestion, bloating, abdominal pain, ulcers and loss of appetite. As an alternative approach, the use of probiotics can help to restore good strains of microbes and intestinal balance and is increasingly becoming popular as the functional food of choice nowadays [3].

In contrast to bacterial probiotics, the potential of yeasts as a source of probiotics is still poorly studied, although they are very important for maintaining the balance of the GI tract such as antagonistic interactions against harmful germs. Some strains (CNCM I-745 and OBS2) of *Saccharomyce*s genus survive through the intestinal tract and are also used industrially as dietary supplements because they contain high mineral content and nutrients [4,5]. To restore and maintain the natural biota in the gastrointestinal tract, probiotic yeast is used as a nutritional supplement, for example in Kefir milk [6,7], cereal-based fermented beverage [8], Red yeast rice [9], maize-based cereals [10] etc. Reductions in symptoms of acute diarrhea and related diseases are observed on consuming *S. boulardii* and *S. cerevisiae* used as pharmaceutical products [11,12]. On the basis of safety and efficacy of the probiotics, many clinical trials and experimental studies strongly propose a place for *S. boulardii* as a biotherapeutic agent for the prevention and treatments of several diseases related to gut [13]. *S. boulardii* is patented yeast and the only probiotic yeast that has been proven very effective in double-blind studies [14]. The yeast *Kluyveromyces marxianus* (S97, S101 and S106) also has been reported to have probiotic properties, such as modulation of the immune response [15,16,17].

The probiotic properties, for example modification of microbial population and aggregation of pathogenic bacteria, are dependent on the mode of action. Some essential probiotic properties include tolerance to bile salts, acidity, body temperature, stomach acid, pepsin and antibiotics. In addition, probiotics should also have desirable attributes, such as good metabolism of sugar, cell surface hydrophobicity, utilization of carbohydrates, autoaggregation and antimicrobial properties. The modes of action of yeast probiotics includes the production of specific substances like phenolic compounds, organic acids, mycocins and dipicolinic acid, as well as competitive adhesion to epithelial receptors, competition for nutrients, modification of the structure and functions of intestinal epithelial cells are documented [10]. Human enzymes cannot digest prebiotic foods, which reach the large intestine intact, so our body needs probiotics for their digestion.

Stress-resistance properties of probiotics were characterized to ascertain their potential in the treatment of various gut-related diseases and their applicability in the food and pharmaceutical industries. Resistance to low pH, tolerance to gastric acidity (pH 2.0–2.5), bile salt resistivity and survival ability at increased temperature are considered as a key functional requirement for probiotics, which enables them to survive during passage through the gastrointestinal tract [18]. They need to survive natural barriers in the gut, including body temperature, low pH and high biliary concentration. The pH in stomach can reach 2.5 for 3 h in fed state due to release of gastric juice. The temperature and pHs also affect production of biomolecules. Resistance to stomach pH and temperature is of great importance in predicting the survival and growth of the potential probiotic strains in the gastrointestinal conditions [19,20] has reported that additional characteristics such as acid resistance, bile-stability and antimicrobial activity against food-borne pathogens also contribute to probiotic properties. Although most of the characteristics tested could be assumed to be strain-dependent, it is difficult to find a strain with all the desirable functional properties, and the selection criteria for potential probiotic candidates could therefore be dependent on the purpose of the product in development.

Yeast also contributes to flavor development in fermented foods and their antagonistic activities toward undesirable microbes are now largely recognized. The antagonistic or antimicrobial property of probiotics involves stimulation of host defense systems, immune modulation, competitive exclusion, production of organic acids or hydrogen peroxide and antimicrobials [14,21]. Some antimicrobial substances produced include acetic acid, lactic acid, formic acid, as well as short chain fatty acids [22,23] and killer toxins or mycocins [23]. Antagonistic yeasts starter cultures contribute to food safety by inhibiting the growth of pathogen during fermentation, and to sensory qualities and shelf-life of the product by inhibiting spoilage microorganisms [21]. Probiotic yeasts also prevent adherence of pathogenic bacteria to the gut mucosa [24,25].

To be considered prebiotics, the food item must be fermentable by probiotics in the gut, be resistant to gastric acidity, and stimulate the growth and activity of the intestinal microbe—related to health and well-being [26]. Honey is an outstanding source of prebiotic. It contains oligosaccharides, which cannot be digested in the small intestine. In the large intestine, it is utilized by the microbes and is a non-dairy product. This provides a huge advantage for those who are allergic to dairy products and lactose-intolerant patients. Like dairy products, raw honey has a symbiotic property, making it an excellent alternative source for pre- and probiotics [27]. The prebiotic property of honey has been examined in mice and different cell cultures. It is found that supplementing media with honey caused a rapid growth of *Bifidobacterium* cells usually found in the intestines and increase the level of healthy bacteria in their guts [28].

Yeast is often associated with sugar-rich environments. Detectable levels of yeasts in very high numbers are also observed in most honey samples [29]. Their identification as probiotics may be useful in the future treatment of various gut-related diseases. The genotypic analysis which is referred to as the process of determining differences in the genetic make-up (genotype) of an individual by the examination of the individual’s DNA sequence by a combination of molecular genetic analysis is commonly employed for identification purposes [30]. In parallel, the phenotypic analyses which are basically the classification of microorganisms based on cell structure, cellular metabolism, or cell components via biological assays and microscopy techniques are conducted [31]. Although *S. cerevisiae* and *S. boulardii* have an almost identical genome sequence, making the genetic basis of probiotic potency an intriguing question to address. It is known that *S. boulardii* produces unusually high levels of acetic acid at 37 °C, which has been reported to strongly inhibit bacterial growth. This unique application of probiotic yeast is due to the presence of an SNP (sdh1 F317Y and whi2 S287 *), responsible for its high acetic acid production [32]. Acquisition of antibacterial activity through production of high acetic acid provides its ecological niche of this probiotic yeast. Thus, the presence of these SNP and acid production could be examined for detection of potential yeast probiotics.

Besides probiotic yeasts, several yeast species are present in the honey samples with a potential use as a cell factory [33,34]. The biological production by yeasts is interesting because of the low-cost investment and less pollution involved. Therefore, yeast is used in diverse applications such as biofuel and biochemical fermentation owing to its high survival ability regarding stress and safety. A large amount of evidence indicates that yeasts could be used as a suitable platform for chemical production of valuable metabolites. Some yeast species can produce xylitol as main product, such as *Scheffersomyces* [35,36], *Kluyveromyces* [37] and *Meyerozyma* [38]. Recently, *Meyerozyma guilliermondii* B12 strain has been the best isolated strain from sugarcane bagasse due to its capability to produce xylitol with 0.30 g xylitol/g xylose consumed [39]. Thus, investigation of yeast species as a potential cell factory for metabolite production or conversion will enable several applications promoting bioeconomy.

Here, the genotypic and phenotypic analyses via sequencing of internal transcribed spacer (ITS) and growth assays on stress-resistance properties were used to ascertain their probiotic and metabolite production potential. As probiotics, they should be resistant to stresses related to the gastrointestinal tract including high temperatures, low pH, bile salt stress and different sugar concentrations [40]. Therefore, we aimed to isolate yeasts from honey samples, domesticated in bee farms of Ratchaburi province in the Western part of Thailand and to characterize for probiotic potential. Secondly, depending on their identification, the second aim was to examine the possibility of using some isolates as a cell factory for production of high valuable metabolites such as acids or xylitol.

## 2. Results and Discussion

### 2.1. Identification of Isolated Yeasts from Honey

**Figure 1:**
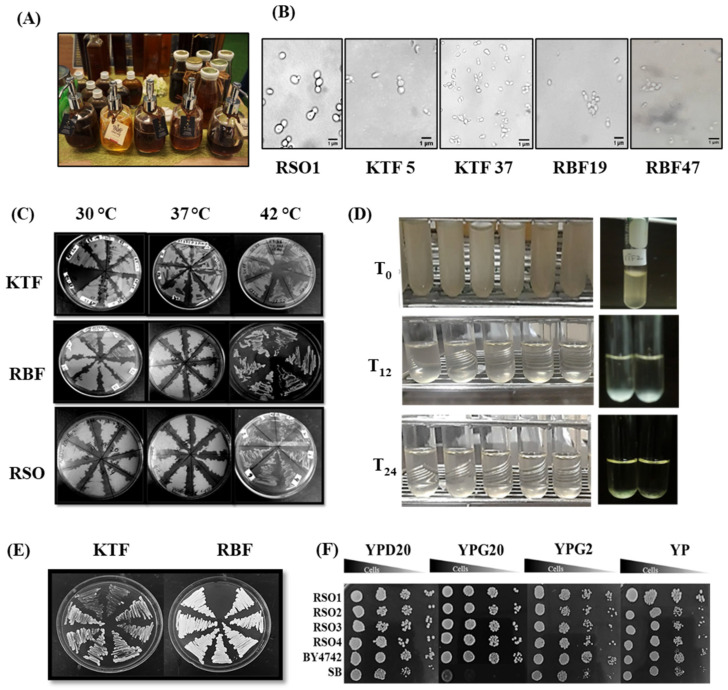
Isolation and characterization of yeast isolates obtained from raw honeybee samples. (**A**) Honeybee products; (**B**) selected images of yeast cells by microscopy; (**C**) thermo-tolerance (30, 37, 42 °C) of KTF, RBF and RSO isolates grown on YPD plates containing 300 mg/L of Ampicillin; (**D**) autoaggregation assays of some selected isolates performed after 2, 4 and 24 h. incubation at 37 °C; (**E**) growth of KTF and RBF isolates on 2% galactose containing plates and (**F**) spot test of RSO isolates, BY4742 and SB strains on Yeast peptone (YP) plates, containing 20% glucose, 20% or 2% galactose. Results were obtained from at least two independent experiments performed in triplicates.

Honey is in fact a rich nutritional source of prebiotics and probiotics and is widely consumed as a household or commercialized product (Figure 1A). Here, one-hundred and four single yeast colonies from different honeybee farms were isolated on YPD plates, containing the antibiotic ampicillin. Different morphology of yeast isolates was observed under the microscope, with KTF yeast isolates exhibiting a white color and a round shape while RBF yeast isolates showed a yellowish color with an oval shape. RSO isolates appeared as the budding yeast with a creamy color (Figure 1B).

Next, natural isolated yeasts were classified with a primer specific-PCR amplification technique to identify the *Saccharomyces* genus using gene specific primers. Briefly, the genomic DNAs of BY4742 *S. cerevisiae* and *S. cerevisiae var. boulardii* Enterol were used as a positive control while *Candida albicans* served as a negative control. Among others, four of the RSO isolates from the Resort honeybee farm (RSO1–4) turned out to be *S. cerevisiae* strains (Table 1). The *S. cerevisiae* found in raw honey of *Trigona apicalis* Smith has been described as a species occurring in the honey of the brood combs and are also transferred into honey in the supers [41]. Indeed, they showed a PCR band with a size of approximately 300 base pairs at the same location as that of *S. cerevisiae* and no band corresponding to the *Candida* genus found (data not shown). Detail on PCR is provided in the Materials and Method section. Afterwards, the ITS sequencing was performed for further use in biochemical tests, excluding *Candida* isolates as they are unsafe for the food industry.

**Figure 2:**
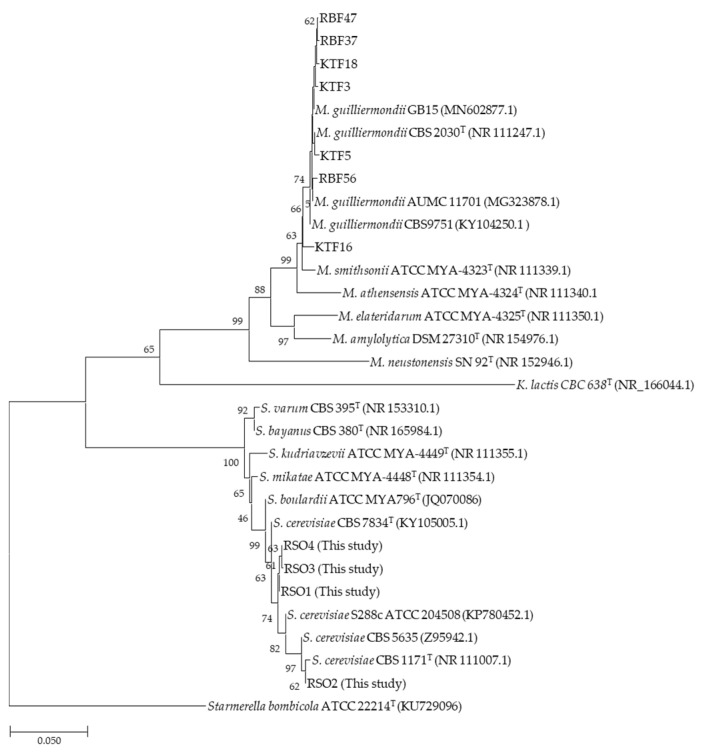
Phylogenetic tree displaying the relationships between some identified yeast isolates (KTF3, KTF5, KTF16, BRF37, BRF47 and BRF56) of *Meyerozyma* and (RSO1, RSO2, RSO3 and RSO4) of *Saccharomyces* obtained from honeybee samples (Table 1). For comparison, based on ITS region sequences from type strains of different species of *Meyerozyma* and Saccharomyces which are retrieved from the literature and NCBI database are shown. The phylogenic tree was constructed using the neighbor-joining method. Bootstrap values were calculated from 1000 replicates. ^T^ symbol indicates the “Type Strain” that is used as a reference.

Some isolates were selectively chosen for further tests based on the phenotypic studies (Table 1) in which some were used for further ITS analysis. Sequencing and analysis of ITS regions using the 18 S (1609–1627) and 26 S (287–266) rRNA identified four and seven yeast isolates belonging to *S. cerevisiae* or *M. guilliermondii* of *A. dorsata* raw honey, respectively (Table 1). For more detail distribution analysis, type strains of former *Meyerozyma* and *Saccharomyces* were used to classify the species of yeast honeybee isolates found. The phylogenetic analysis of these yeast isolates was performed using 10 ITS region sequences and the phylogenetic tree is shown (Figure 2). They were identified into two genus, representing honeybee isolates while the ATCC 8585 represents the type strain of *K. lactis* (KU729077.1) [32] that is also widely employed as a probiotic yeast and is clinically relevant [42]. Some identified yeast isolates (RBF37, RBF47, RBF56, KTF3, KTF5 and KTF16) were closely linked to the type strain of *M. guilliermondii* CBS 2030^T^ (NR 111247.1) [43] while some former type trains of *Meyerozyma* species including *M. smithsonii* ATCC MYA-4323 (NR 111339.1) [43], *M. athensensis* ATCC MYA-4324 (NR 111340.1) [43], *M. elateridarum* ATCC MYA-4325 (NR 111350.1) [43], *M. amylolytica* DSM 27310 (NR 154976.1) [44] and *M. neustonensis* SN 92 (NR 152946.1) were also displayed [45]. *M. guilliermondii* has previously been isolated from honey samples of *A. mellifera*, *A. dorsata*, and *Tetragonula pagdeni*. It is widespread and unlikely to be specifically related to a host insect [46]. Additionally, *Meyerozyma* is also known as the new genus assigned from *P. guilliermondii* and *P. caribbica* [19,47]. In second group, type strains of *Saccharomyces* spices consist of *S. varum* CBS 395 (NR 153310.1) [48], *S. bayanus* CBS 380 (NR 165984.1) [49], *S. kudriavzevii* ATCC MYA-4449 (NR 111355.1) [43], *S. mikatae* ATCC MYA-4448 (NR 111354.1) [43], *S. boulardii* ATCC MYA796 (JQ070086) [50], *S. cerevisiae* CBS 7834 (KY105005.1) and *S. cerevisiae* CBS 1171 (NR 111007.1) [43] are shown. These remaining yeast isolates (RSO1–4) belonged to this group in which two isolates RSO3 and RSO4 are closely linked and related to RSO1. Furthermore, this group is a sister taxon of a subgroup containing RSO2 which is closely linked to the strain type of *S. cerevisiae* CBS 1171. The RSO1–4 isolates were all belonged to *S. cerevisiae* (Figure 2), which is preferred for use in fermentation [51]. *S. var. boulardii* and *S. cerevisiae*, generally recognized as safe (GRAS), are commercially used as probiotics.

### 2.2. Determination of Thermo-, Low PH and Bile Salt Tolerance of Yeast Isolates

Thermotolerance, resistance to low pH, tolerance to gastric acidity (pH 2.0–2.5), and bile salt resistance are considered key functional requirements for probiotics, which enable them to survive during passage through the gastrointestinal tract [40]. Thermotolerance is one of several important properties as probiotics need to survive natural barriers in the gut, including body temperature, low pH, and high biliary concentration. First, yeast isolates were subjected to high temperatures. In parallel to the genotypic analysis using gene specific PCR and ITS analysis, phenotypic studies on stress tolerance were also conducted. Ninety-four isolates out of one-hundred and four isolates were able to grow at 30 °C and 37 °C, while some grew at 42 °C (data not shown). Those isolates were then selected for their ability to survive at low pH, since the pH in the stomach can reach the value of 2.5 for 3 h in a fed state due to release of gastric juices [14]. Some of the yeast isolates, as shown in Table 1, with viable growth under a pH of 2 were selected for bile salt tolerance. The survival ability of yeast at pH 2.5 has also been previously reported [52]. Resistance to low pH is observed in yeast cells, indicating they may have developed molecular mechanisms to respond to adverse conditions or changes in cellular metabolism [53].

After, bile salt tolerance of microorganisms is used as a selective criterion for selection of potential probiotics. The bile resistance of microorganisms is related to bile salt hydrolase activity, which contributes to reduction in the toxic effect of conjugated bile. Since a concentration of 0.3% bile salts is critical in screening for human probiotics, growth of yeast isolates was examined in 0.3% bile salt, pH 2.5, and at 37 °C, as described by [40] with some modification. A total of 58 yeast isolates survived in the presence of bile salt. The RBF isolates (RBF1–12) showed the lowest tolerance to bile salt and were excluded from further tests (Table 1). KTF isolates (KTF 1, 2, 3, 5, 6, 14, 15, 16, and 18) and RSO isolates (RSO1–4) showed high survival under test conditions (Table 1). Some yeast strains belonging to *Trichosporon cutaneum*, *Candida rugosa,* and *Candida lambica* are resistant to up to 2% bile concentrations, indicating that they could survive bile toxicity during their passage through the gastro-intestinal system [54]. Nevertheless, most characteristics tested could be assumed to be strain dependent. It is difficult to find a single strain with all the desirable functional properties, and the selection criteria for potential probiotic candidates could therefore be dependent with the purpose of the product development. Characteristics of the strains, such as thermotolerance, acid resistance, bile-stability, and antimicrobial activity against food-borne pathogens, may contribute to development of health products with potential probiotic properties.

### 2.3. Autoaggregation Ability of Yeast Isolates

Another desirable characteristic of a potential probiotic microorganism is the ability to form cellular aggregates, since aggregates can increase the microbial adherence to the intestine, thus providing advantages in the colonization of the GI tract [55]. Autoaggregation, sometimes also called autoagglutination or flocculation, is the formation of yeast or bacterial clumps that settle at the bottom of culture tubes. It is macroscopically observed. The RSO1–4, some KTF and RBF isolates formed multicellular clumps that eventually settle at the bottom of culture tubes (Figure 1D). However, some isolates showed continuous growth and cloudiness, indicating that they were unable to settle down into the tubes or unable to attach to the surface or with each other (Figure 1D).

Autoaggregation is generally mediated by self-recognizing surface structures, such as proteins and exopolysaccharides, which collectively are termed autoagglutinins. However, despite it being a widespread phenomenon, in certain cases the function of autoaggregation is poorly understood. Some evidence shows that aggregating bacteria or yeast cells are protected from environmental stresses or host responses, which is considered one of the probiotic strain’s properties. pH changes in the medium as a result of growth-coupled ion exchange or production of organic acid, ethanol or antimicrobial compounds such as killer toxins also affect antagonism of microorganisms by yeasts [56]. Here, the effect of pH on stabilization/destabilization of yeast and cell structure in the aggregation test was done at a neutral pH of 7 and the effect of pH at different time points was minimized, via normalization to time zero. Notably, the preparation process may have diffusion limitation although the optimum condition used was as provided in the Methods. The results of this assay revealed that some yeast isolates displayed a desirable autoaggregation property, with an autoaggregation rate above 80% (strong) (Table 1). There are reports of *S. cerevisiae* isolates with an autoaggregation percentage of 85%. Tested isolates with promising autoaggreagtion ability, such as the RSO strains with 100% autoaggregation ability (Table 1), were selected for the carbohydrate utilization assay.

### 2.4. Utilization of Sugars

Galactose is a simple sugar that is normally transformed in the liver before being used up as energy. This sugar is quite abundant in human diets and helps in many functions. As galactose is a precursor to glucose production, it is an important nutrient that provides energy. Galactose is also important in human metabolism, with an established role in energy delivery. There is strong evidence of the potential therapeutic benefits of galactose [57]. The high concentration of 2% galactose present is the main reason for the growth of galactose positive and non-fermenting yeasts in yogurt [58]. KTF and RBF isolates were cultured using different concentrations of galactose at 37 °C on plates, and they presented good survival in comparison with galactose non-assimilating commercial strain *S. boulardii* (Enterol) (Figure 1E and Table 1). *S. boulardii* cannot utilize galactose in abundant which is one of the drawbacks of this probiotic yeast [59]. On the other hand, *S. cerevisiae* can grow in galactose to high numbers (Figure 1E and Table 1). As expected, the *S. cerevisiae* RSO isolates showed normal growth on galactose-containing media (Figure 1E and Table 1). Growth on galactose is a favorable property of yeast isolates as they can be utilized in food and drinks.

### 2.5. Organic Acid Production by S. cerevisiae RSO Strains

**Figure 3:**
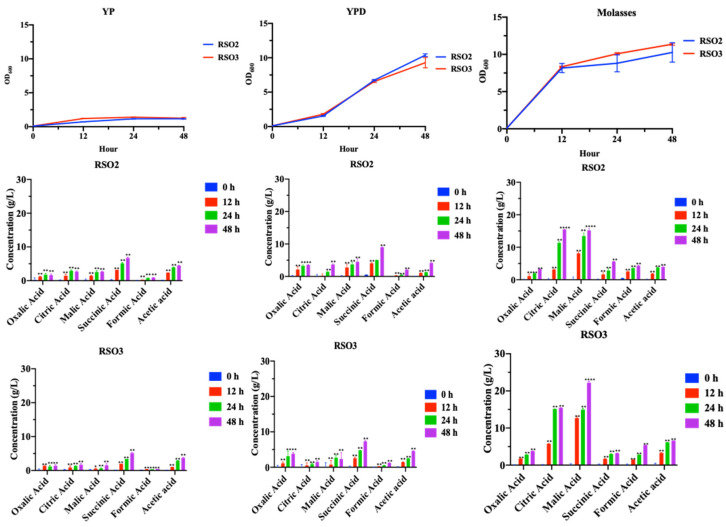
Organic acids production of isolated *S. cerevisiae* RSO2 and RSO3 strains from fermentation of 5% sugarcane molasses. Concentrations of oxalic, citric, malic, succinic, formic and acetic acids (g/L) were determined by HPLC-UV or RI analysis and expressed as a percentage of the maximum concentration observed for each metabolite. Results were obtained from at least two independent experiments performed in triplicate. Significant differences of organic acid concentrations produced at different timepoints in comparison with the initial timepoint (at 0 h) were determined by one-way ANOVA with the Tukey HSD method (*, *p* < 0.05; **, *p* < 0.01; ****, *p* < 0.0001). Error bars indicate SD.

Moreover, some studies have suggested that the proper use of feed additives containing acids during stress could prevent disease in animals and enhance growth and health status [23]. Organic acids are no longer function as simple acidifiers, but rather as growth promoters and potential antibiotic substitutes due to their beneficial action on the gastrointestinal tract [23]. Since the *S. cerevisiae* RSO strains displayed ability to survive under low pHs and yeast probiotics often produce high level of acetic acid, the capacity to produce acids was also addressed using glucose and non-conventional carbon source. Cells of *S. cerevisiae* RSO2 and RSO3 strains were selectively cultured in broth media of YP, YPD or YPM (5% sugarcane molasses) for 48 h. The yields of different acids including oxalic, citric, malic, succinic, formic and acetic acids were examined via HPLC analysis. Importantly, some of these organic acids represent intermediary products of the tricarboxylic acid cycle (TCA) which provides energy source while others function as antimicrobial substance [53]. As shown, using sugarcane molasses as a carbon source resulted in increase in production of several high value acids such as citric, malic and succinic acids although the capacity of acid production varied among the RSO strains (Figure 3).

Further studies should seek to evaluate the impacts of different acids yeast produced at different phases of life during feeding for probiotic application. Next, using 5% molasses, the RSO2 and RSO3 produced the similar level of oxalic acid at 3.28 and 3.78 g/L and citric acid at 15.46 and 15.43 g/L, respectively. The RSO3 strain produced 22.21 g/L and 3.21 g/L while RSO3 produced 15.19 and 5.58 g/L of malic and succinic acids, respectively (Figure 3). Formic and acetic acids were also produced from fermentation of molasses at lower levels with the maximum yields of 4.31 and 3.97 g/L by RSO2 and 5.39 and 6.85 g/L by RSO3, respectively (Figure 3). Overall, results indicated that sugarcane molasses are a suitable substrate for acid production and, these *S. cerevisiae* RSO2 and RSO3 strains are promising for production of several valuable acids especially citric and malic acids. Significant levels of citric and malic concentrations were obtained. They appeared to be maximally produced at 48 h of fermentation of molasses (Figure 3). Previously, using beet molasses, 52.3 g/L of citric acid is achieved at the condition in which the initial sugar concentration is 140 g/L at pH 5.0, 30 °C by *Aspergillus niger* [60]. *Candida tropicalis* could and *Yarrowia lipolytica* produce over 132.2 and 66.2 g/L of citric acid using optimum nitrogen sources, respectively [61]. Similarly for malic and succinic acids, several yeast strains are engineered or their growth conditions are optimized to improve the production [35,62]. Fermentation of cane molasses and corn steep liquid by a strictly anaerobe bacteria *Anaerobiospirillum succiniciproducens* which produces high level of succinic acid [63]. However, it fails to thrive at the low pH value of 6.5, thereby requiring an additional step of neutralization of the produced acid. Discovery of yeast strains such as RSO allows for acid production at a relative low pH. Following a metabolic engineering approach to develop novel strains, these yeast strains may meet the process requirements for converting biomass feedstock into the desirable product.

### 2.6. Xytitol Production by M. guilliermondii MX Strain

**Figure 4:**
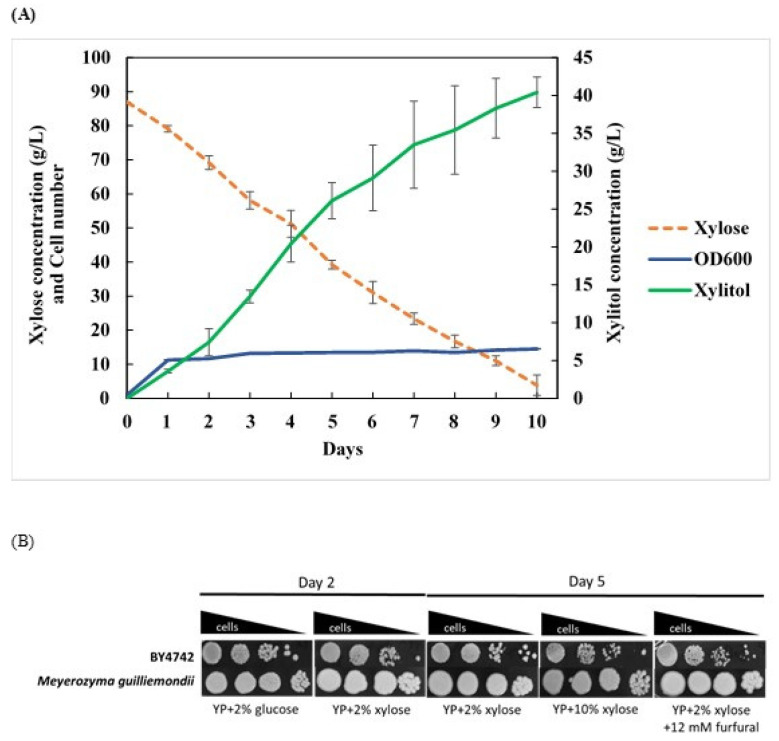
Xylitol production during fermentation of xylose by a selected *M. guilliermondii* isolated from the *Apis dorsata* Fabricius raw honeybee sample. (**A**) The growth curve indicated the optical density of cells at OD_600_ and changes in xylose and xylitol concentration (g/L) of *M. guilliermondii* during 10 days of fermentation with 10% (*w*/*v*) xylose at 30 °C. (**B**) Survivability of *M. guilliermondii* and *S. cerevisiae* BY4742 on YP plates containing 2% (*w*/*v*) glucose, 2% or 10% (*w*/*v*) xylose as a carbon source with or without 12 mM lignocellulosic inhibitor furfural. Results were obtained from at least two independent experiments performed in triplicates.

For xylitol production, a continuation study of carbon source utilization by isolated yeasts was investigated using xylose. *M. guilliermondii* has been a subject of interest in several studies with broad applications [64]. For example, it is employed in the production of riboflavin [65], xylitol [39], and enzymes [66] as well as biofuel [67]. Here, the isolated *M. guilliermondii* MX(L27/152) was also tested on the ability to utilize xylose as a carbon source. Interestingly, it could consume almost all of xylose in 10 days with xylose consumption of 0.35 g/L/h, xylose consumption rate 0.10 g/g CDW/h and the producing highest xylitol production of 40.40 g/L or xylitol yield 0.49 g/g of consumed xylose (Figure 4A). The xylitol production capability of *M. guilliermondii* isolated from honeybee samples was better than the previous report of the *M. guilliermondii* B12 strain isolated from sugarcane bagasse with the maximum xylitol yield of approximately 0.32 g/g [68]. Recently, the overexpression of *XYL1* and knockout of *XDH1* genes encoding in *M. guilliermondii* ATCC6260 promotes xylitol production with specific productivity of 0.27 g/g xylose [38]. However, the *M. guilliermondii* MX strain did not show accumulation of extracellular glycerol when exposed to high-salt stress as previously described [69]. Additionally, the newly isolate *M. guilliermondii* MX strain showed high tolerance to osmotic and oxidative stresses on xylose plates containing inhibitors such as furfural when compared with the laboratory *S. cerevisiae* BY4742 strain (Figure 4B). This enables the potential application of isolated yeast strain for a conversion of xylose to xylitol or other bioproducts from lignocellulosic biomass.

Additionally, with respect to maintenance, stability as well as on activity of these potential yeast probiotics and a cell factory, the effect of was normal at all studied pH values and temperatures tested. There was no significant decrease in activity or growth observed. The effect of temperature on the relative activity of yeast and cell structures may also affect the stabilization although these yeast isolates appeared to be stable at the temperature range between 30 and 37 °C. The effects of these parameters, if any, have to be evaluated in detail in future studies; nevertheless, the data presented that these yeast isolates could grow under specific parameters or conditions tested (Table 1 and see also Materials and Methods).

To this end, these new yeast isolated strains display promising potential for applications in the biotechnological industry that serves as a basis for other industries. For examples, acid has a wide range of food, pharmacy, biopolymers, coatings, green solvents, and plasticizers as a precursor for the synthesis of other value-added products. Moreover, the global acid- and bio-based chemical market is expected to double during the forecasted period of 2016–2025. Although, most compounds are mainly produced via a chemical process from petroleum-based material that is expensive and causes serious pollution problems. Engineering industrial cell factories to effectively yield a desirable product while minimizing industrially relevant stresses is usually the most challenging task in the development of industrial production of biochemicals using the process of microbial fermentation.

A microbial cell factory is an approach to bioengineering that considers microbial cells as a production facility in which the optimization process generally depends on metabolic engineering. Some limitations such as complex regulation or poor gene expression are challenges so understanding complex biological networks is essential. The advance of omics technologies, based on a holistic view of the molecules that aims primarily at the universal detection of genes, mRNA, proteins, and metabolites in a specific biological sample, become necessary. A complex biological system can be understood more thoroughly if considered as a whole and omics technology can be useful to guide synthetic biology design and impact metabolic engineering towards the goal of developing improved yeast cell factories. Use of omics analysis of these isolated yeast stains will enable better understanding of complex biological networks and construction of yeast cell factories for high value biochemical production.

## 3. Materials and Methods

### 3.1. Sample Collection and Yeast Isolation

Three raw honeybee samples of *Apis dorsata* Fabricius and *Trigona apicalis* Smith taken from districts of Ratchaburi province of Thailand at Krung Thong Dee (KTF), Rang Boa (RBF) and Resort (RSO) farms were collected in the year 2019. They were diluted ten times in sterile water for isolation of yeast by the spread plate technique of [70]. One hundred microliters of diluted honeybee samples were spread on Yeast extract Peptone Dextrose (YPD) plates, containing 2% (*w*/*v*) glucose, 2% (*w*/*v*) peptone, 1% (*w*/*v*) yeast extract, 2% (*w*/*v*) agar and antibiotics. The Kirby Bauer method was used to examine the antibiotic resistivity. Briefly, a filter paper disc was loaded with the specific amount of antibiotic, 0.3 mg/mL of ampicillin, and the zone of inhibition was obtained. Cells were incubated at 30 °C for 3–4 days prior to being examined under a light microscope to observe their morphology. A single colony of yeast isolates were re-steaked on YPD agar. Collections of yeasts isolates were stored in 20% (*v*/*v*) of glycerol stock containing some YPD at −80 °C for further study.

### 3.2. Sequence Analysis of the ITS Regions

First, the genomic DNAs of yeast isolates was extracted by the phenol:chloroform:isoamyl method. The genomic DNAs of BY4742 *S. cerevisiae*, Enterol *S. cerevisiae var. boulardii* and *Candida albicans* as served positive and negative controls, respectively. The specific primers corresponding to *Saccharomyces* genus included F-C871: 5′-GCGCTTTACATTCAGATCCCGAG and R-C8672: 5′-TAAGTTGGTTGTCAGCAAGATTG [71] as described by Muir et al. (2011), or to a *Candida* gene included F-C7112: 5′-TTAAGTCCCTGCCCTTTGTA and R-C7113: 5′-GCATTCCCAAACAACTCGACT T [72] were used. PCRs were performed and the products were run on the gel to identify if any *Saccharomyces* or *Candida* isolates were present. Afterwards, sequencing and analysis for ITS regions of the 18 S (1609–1627) and 26 S (287–266) rRNA were performed for the identification of microorganisms [42,73]. The genomic DNA was extracted by the phenol:chloroform:isoamyl method and amplified by PCR with Q5 High-Fidelity DNA polymerase (M0491, NEB) using the primers (ITS-C7112 5′-TTAAGTCCCTGCCCTTTGTA-3′ and ITS-C7113 5′-GCATTCCCAAACAACTCGACT-3′). The PCR master mix included 200 μM of dNTPs, 100 pmol of forward or reverse primers, 100 ng of genomic DNA template, 1 mM MgCl_2_, 1X reaction buffer, 2.5 U of High-fidelity DNA polymerase in a total volume of 50 μL of DI water. The program for amplification was as follows: initial denaturation 98 °C, 30 sec; followed by 30 cycles at denaturing 98 °C, 10 s; annealing 54 °C, 20 s; extension 72 °C, 60 s; and final extension 72 °C, 2 min. The total reaction volume was 50 μL per tube. The PCR products were run on 1% agarose gel. Fifty μL of 10 μM PCR products were sent to Sanger sequencing service. The sequencing primers used were ITS-C7114 5′-TCCGTAGGTGAACCTGCGG-3′ and ITS4-C7115 5′-TCCTCCGCTTATTGATATGC-3′ A sequence similarity analysis using information deposited in the NCBI databases was performed. The DNA amplification comparison technique was described by [74,75].

### 3.3. Phylogenic Tree Analysis Check

Sequences for all strains identified to the species level were used in the blast analysis with GenBank with the accession numbers. Sequences were edited and manually corrected with Molecular Evolutionary Genetics Analysis (MEGA) software, version 10.1 [76]. Multiple-sequence alignment was performed by ClustalW 1.8. Phylogenetic trees were constructed by the neighbor-joining method using MEGA 10.

### 3.4. Growth Assays at Increased Temperatures, Low PHS, and 0.3% of Bile Salt

In order to analyze cell ability to grow under the stimulated gastrointestinal condition, growth of isolated yeast strains was examined at pH 2 to 2.5, 37 degrees Celsius and 0.3% bile salt media. Cells were incubated at different temperatures (30, 37 and 42 °C) to check for tolerance. The bile salt (Sigma-Aldrich, St. Louis, MO, USA) stock was dissolved in yeast synthetic or yeast nitrogen base YNB media containing 6.7 g/L Yeast nitrogen base and 10 g/L of glucose buffered at pH 7, 5.4 or 2.5. Cells were incubated in 96-well plates (Bioscreen C, Labsystem, Helsinki, Finland). Changes in optical density of cells were measured every hour from 0 to 56 h. Harvesting of cells was done by centrifugation for 10 min at 3000 g at room temperature. Yeast growth was determined as the area under the growth curve (OD_600_ × h) which were obtained from the bio screen data. Growth of yeast cells in 0.3% (*w*/*v*) bile salt (Sigma-Aldrich, St. Louis, MO, USA) YNB at pH 2.5 was compared with growth at pH 5.4 and pH 7, positive controls.

### 3.5. Autoaggregation Assay

Cultivation of yeast isolates was performed at 37 °C for 24 h in 5-mL YPD broth. Cells were centrifuged washed and resuspended in phosphate buffer saline (PBS) at pH 7. The cell suspension was agitated by vortexing in 3 mL of the same solution and an aliquote of 1 mL was transferred to a disposable plastic cuvette (BrandTech 759165). Determination of autoaggregation was made at 12 h and 24 h of incubation at 37 °C by the optical density (OD_600_ nm) using a spectrophotometer as described by [77]. To obtain the percentage of autoagregation, the formula used: % Autoaggregation = [1 − (A_t_/A_0_)] × 100 where A_0_ is at time zero and A_t_ was the absorbance at time of 2, 4, or 24 h.

### 3.6. Carbohydrate Utilization

Spot test was conducted for the carbohydrate utilization in which serial dilutions of yeast isolates (RSO1–4), *S. boulardii* (SB) and *S. cerevisiae* (BY4742) were spotted onto YP plates with different galactose at concentrations of 20 or 2 g/L, glucose at concentrations of 20 or 2 g/L or YP without any carbon sources as the control. Other yeast samples were streaked on media containing different galactose concentrations. For xylose utilization, the wild-type *S. cerevisiae* BY4742 and *M. guilliermondii* strains were grown in 3 mL of YPD broth, incubated at 150 rpm at 30 °C overnight. Cells were serially diluted to an OD_600_ of 0.1, 0.01, 0.001, and 0.0001. An amount of 3 μL of serial diluted cells were spotted on the YPX agar plates, containing different concentrations of xylose at 2 to 10% (*w*/*v*) with or without 12 mM Furfural and incubated at 30 °C for 2–5 days.

### 3.7. Metabolite and Cell Density Analyses

For acid detection, yeast isolates *S. cerevisiae* (RSO2 and RSO3) were grown overnight in YPD, with shaking at 30 °C and 150 rpm. Then, cells were regrown an initial optical density at 600 nm (OD_600_) of 0.1 to 0.6, inoculated into 50 mL of YP, YPD or YPM broth containing either no glucose, 2% glucose or 5% sugarcane molasses, respectively, at 30 °C with shaking at 150 rpm. Culture broth was collected at times of zero, 12, 24 or 48 h. To measure concentrations of acid by HPLC using an Aminex HPX-87H ion-exchange column (300 × 7.8 mm i.d.) (Bio-Rad, Hercules, CA, USA), 5 mM H_2_SO_4_ as a mobile phase, the flow rate of eluent (5 mM H_2_SO_4_) at 0.6 mL/min. The column temperature was maintained at 65 °C. Detection was performed with a PDA detector (Shimadzu, Nakagyo-ku, Kyoto, Japan,). Acid identified in samples was based on a retention time using acid standards (Biorad, Hercules, CA, USA) and the concentration was determined based on peak area. For xylitol production, a full loop of yeast *M. guilliermondii* was cultured in 50 mL of YPD broth, incubated at 150 rpm at 30 °C overnight. Total cells were resuspended in distilled water and diluted to an OD_600_ of 1.0 (3 × 10^7^ cells/mL), then transferred into 50 mL of YPX10 broth containing 100 g/L of xylose as a sole carbon source. Cell samples (1.5 mL) were harvested from day 1 to 10 for measurement of optical density at 600 nm. Metabolite samples were analyzed for concentrations of xylose, xylitol, glycerol, and ethanol by using HPLC (Shimadzu, Nakagyo-ku, Japan, Kyoto) with an Aminex HPX-87H ion-exchange column (300 × 7.8 mm i.d.) (Bio-Rad, Hercules, CA, USA). The elution was performed with 5 mM H_2_SO_4_ at a flow rate 0.6 mL/min and the column temperature was 65 °C. Detection was performed by refractive index (RI) measurement (Waters, 2414 RI detector). Cell concentrations were determined from the optical density measurements at 600 nm using a spectrophotometer.

### 3.8. Statistical Analyses

Data were analyzed and presented as the mean standard deviation (SD) for the indicated number of at least two independently performed experiments carried out in triplicate. The metabolite data were analyzed using a SPSS software, version 26.0 (SPSS, Inc., Armonk, NY, USA). Analysis of variance (ANOVA) was applied for significant differences of metabolite using timepoints as a factor.

## 4. Conclusions

In summary, a total of one hundred and four yeast isolates were obtained from honeybee samples collected from three diverse locations in the Western part of Thailand. Growth under the stimulated gastrointestinal condition revealed fifty-eight isolates (33 from RBF, 21 from KTF, and 4 from RSO honeybee collection) with the ability to grow at low pH of 2–2.5 and to tolerate a high temperature of 37 °C at a 0.3% (*w*/*v*) bile salt concentration. In general, yeasts linked with food have been found to survive under such conditions. All isolates that pass the above criteria exhibit a different autoaggregation capacity between 70% and 100% after 24 h of incubation at 37 °C and a pH of 7. Fourteen isolated strains show a greater than 80% autoaggregation ability. Genotypic analysis using specific primers and ITS sequencing identified fifty-four as non-*Saccharomyces* and four isolates as *S. cerevisiae*. The isolates RSO2 and RSO3 display good ability to produce the antimicrobial acetic acid and other valuable acids using glucose or molasses as a substrate, respectively. Among them, ten yeast isolates belonging to *M. guilliermondii* display the ability to produce high level of valuable sugar xylitol. Thus, yeast isolates of honeybee samples show high potential as probiotics and cell factory for production of valuable acids and xylitol. The metabolite production by yeasts has a promising future in agro-industrial by-product utilization for a sustainable development of bioeconomy. Further studies are required prior to implement yeast isolates as potential probiotics for health and well-being or, alternatively, to be exploited as a cell factory.

## Figures and Tables

**Table 1:** Summarized characteristics of isolated yeast strains (RSO, KTF, RBF), *S. cerevisiae* BY4742, *S. var. boulardii* (SB). The survival ability under different temperatures and pHs, growth in the presence of 0.3% bile salt, autoaggregation ability, galactose utilization and strain identification are shown. a–d: Different superscript letters in the same column indicate statistical differences in each strain at the level of *p* ≤ 0.05 as measured by Tukey’s test. All the values were represented as mean SDs of three independent replicates from at least two independent experiments. The symbols (√) or (-) indicated an ability or inability of isolates found under tested conditions, respectively.

No.	Isolate Name	Temp. 30 °C	Temp. 37 °C	Temp. 42 °C	PH 2.5	PH 2.0	0.3% Bile Salt	Auto- Aggregation Ability (%)	Galactose Utilization Ability	Genus/Species Identification
1	RSO1	✓	✓	✓	✓	✓	✓	100 ^a^	✓	*Saccharomyces cerevisiae*
2	RSO2	✓	✓	✓	✓	✓	✓	100 ^a^	✓	*Saccharomyces cerevisiae*
3	RSO3	✓	✓	✓	✓	✓	✓	100 ^a^	✓	*Saccharomyces cerevisiae*
4	RSO4	✓	✓	✓	✓	✓	✓	98 ^a^	✓	*Saccharomyces cerevisiae*
5	KTF1	✓	✓	-	✓	✓	✓	80 ^b^	✓	Non- *Saccharomyces*
6	KTF2	✓	✓	-	✓	✓	✓	86 ^b^	✓	Non- *Saccharomyces*
7	KTF3	✓	✓	✓	✓	✓	✓	91 ^a^	✓	*Meyerozyma guilliermondii*
8	KTF5	✓	✓	✓	✓	✓	✓	95 ^a^	✓	*Meyerozyma guilliermondii*
9	KTF6	✓	✓	✓	✓	✓	✓	73 ^c^	✓	Non- *Saccharomyces*
10	KTF16	✓	✓	✓	✓	✓	✓	78 ^c^	✓	Non- *Saccharomyces, Meyerozyma*
11	KTF18	✓	✓	✓	✓	✓	✓	72 ^c^	✓	*Meyerozyma guilliermondii*
12	RBF19	✓	✓	-	✓	✓	✓	89 ^b^	✓	Non- *Saccharomyces*
13	RBF20	✓	✓	-	✓	✓	✓	68 ^d^	✓	*Meyerozyma guilliermondii*
14	RBF27	✓	✓	-	✓	✓	✓	81 ^b^	✓	Non- *Saccharomyces*
15	RBF37	✓	✓	✓	✓	✓	✓	52 ^d^	✓	*Meyerozyma guilliermondii*
16	RBF47	✓	✓	✓	✓	✓	✓	70 ^c^	✓	*Meyerozyma guilliermondii*
17	RBF56	✓	✓	✓	✓	✓	✓	88 ^b^	✓	*Meyerozyma guilliermondii*
18	BY4742	✓	-	-	✓	✓	✓	100 ^a^	✓	*Saccharomyces cerevisiae*
19	SB	✓	✓	-	✓	✓	✓	96 ^a^	-	*Saccharomyces var. boulardii*

## Data Availability

Data is contained within the article.
